# Promoter Methylation of Four Tumor Suppressor Genes in Human Papillary Thyroid Carcinoma

**DOI:** 10.30699/ijp.2019.94401.1922

**Published:** 2019-09-22

**Authors:** Fatemeh Khatami, Bagher Larijani, Ramin Heshmat, Shirzad Nasiri, Hiva Saffar, Gita Shafiee, Azam Mossafa, Seyed Mohammad Tavangar

**Affiliations:** 1 *Chronic Diseases Research Center, Endocrinology and Metabolism Population Sciences Institute, Tehran University of Medical Sciences, Tehran, Iran*; 2 *Endocrinology and Metabolism Research Center, Endocrinology and Metabolism Clinical Sciences Institute, Tehran University of Medical Sciences, Tehran, Iran*; 3 *Department of Surgery, Tehran University of Medical Sciences, Shariati Hospital, Tehran, Iran*; 4 *Department of Pathology, Shariati Hospital, Tehran University of Medical Sciences, Tehran, Iran *

**Keywords:** Tumor suppressor genes, Methylation, Papillary thyroid cancers

## Abstract

**Background & Objective:**

Papillary thyroid cancer (PTC) is considered to be the most common type of thyroid malignancies. Epigenetic alteration, in which the chromatin conformation and gene expression change without changing the sequence of DNA, can occur in some tumor suppressor genes and oncogenes. Methylation is the most common type of epigenetic alterations that can be an excellent indicator of PTC invasive behavior.

**Methods:**

In this research, we determined the promoter methylation status of four tumor suppressor genes (*SLC5A8, RASSF1, MGMT, *and* DNMT1)* and compared the results of 55 PTC cases with 40 goiter patients. For methylation, we used the methylation-sensitive high resolution melting (MS-HRM) assay technique. The resulting graphs of each run were compared with those of 0%, 50%, and 100% methylated controls.

**Results:**

Our data showed that the promoter methylation of *SLC5A8*, *Ras association domain family member 1(RASSF1)*, and *MGMT* were significantly different between PTC tissue and goiter with P-value less than 0.05. The most significant differences were observed in *RASSF1*; 77.2% of hyper-methylated PTC patients versus 15.6% hyper-methylated goiter samples (*P*<0.001).

**Conclusion:**

RASSF1 promoter methylation can be a PTC genetic marker. RASSF1 promoter methylation is under the impact of the methyltransferase genes (DNMT1 and MGMT), protein expression, and promoter methylation.

## Introduction

Endocrine tumors include thyroid, adrenal, pancreas, parathyroid, and pituitary glands (1). Thyroid cancers are classified into four main types: papillary thyroid cancer (PTC), follicular thyroid cancer (FTC), medullary thyroid cancer (MTC), and anaplastic thyroid cancer (ATC) (2, 3). PTC is the most common form of well-differentiated thyroid cancer (1.0%–1.5% new cases per year) with growing incidence over the last three decades all over the world (4-8). PTC is typically an asymptomatic disease and it is identified through the mass in the anterior neck of patients usually when they are in their thirties and forties (9-11). PTC cells can occasionally migrate to the adjacent lymph nodes and rarely to distant organs; thus, adjacent and distant metastasis to the lungs and bones can be seen in metastatic form of PTC (12, 13). Nowadays, fine needle aspiration (FNA) is a common test for evaluating thyroid nodules, but it can be reported as the uncertain in some rare cases (14, 15). Accordingly, finding some genetic biomarkers for classifying malignant and benign cases before metastasis could be an essential measurement for both thyroid cancer patients and clinicians (16-19). In thyroid malignancies, the most important genetic and epigenetic alterations start their functions through activating metabolic pathways like *mitogen-associated protein kinase (MAPK)/extracellular signal-regulated kinase (ERK)* (20-22). Genetic and epigenetic changes of the genome can result in protein expression alterations like *EGFRvIII*, *CD56, P63, CK19, *estrogen receptors (*ERs*), and *Survivin* that are usually checked by immune-histochemical (IHC) studies (23-26). Histopathology is the microscopic study of targeted surgically removed tissue and for accurate diagnosis of cancer and other diseases, histopathological examination of samples is required (27-31). IHC is a useful method for determining the exact origin of tumor cells and sometimes discrimination between non-neoplastic disorders (32-35). Common histopathology and clinical features can be used for PTC and other papillary cancer types; moreover, some additional genetic and epigenetic biomarkers can support them (36). In fact, genetic and epigenetic biomarkers can fill the gap of exact diagnosis through imaging (ultrasound technology) and cytology, as the usual detection methods (37-39). Some genetic markers are mutations, polymorphisms, amplifications, and translocations, and epigenetic markers addition to microRNAs (34, 40). Contrary to genetic modifications that alter the sequence of genes constantly, the methylation is resulting in the alteration of gene expression patterns without changing the DNA sequence in a reversible manner (41, 42). Epigenetic silencing through aberrant DNA methylation of tumor suppressor genes can bring devastating consequences and cause human cancer formation (42, 43). Hyper-methylation of several tumor suppressor genes (*TSHR, ECAD, SLC5A8, DAPK, TIMP3,* and *RARB2*) are linked to the aggressive features of PTC (27, 44-47). Methylation status is mostly reported as the Methylated (M) or Unmethylated (U) so the methylation quantity is not available. Unfortunately, the common use of non-quantitative methylation detection method cannot represent the exact methylation in promoter region of reported hyper-methylated loci. Moreover, non-quantitative methylation detection method is prone to the inclusion of false positive results (48). Furthermore, the failure to quantify methylation incorrectly assumes homogeneity of stages and the significance of all detected methylation (49). Thanks to the new approach of promoter methylation quantification based on high resolution melting (HRM), now it is possible to determine the quantity of methylated cytosine in CpG dinucleotide (CpG islands) (50-53). Assessment of DNA methylation quantity can be a critical factor for the identification, development, and application of methylation-based biomarkers in cancer. This study aims to identify DNA methylation quantity of four tumor suppressor genes using the methylation-sensitive high resolution melting (MS-HRM) assay technique. 

## Materials and Methods

**Tissue** **Samples**

This study was approved by the Research Ethics Committee of the Endocrinology and Metabolism Research Institute, Tehran University of Medical Sciences (IR.TUMS.EMRI.REC.1395.00114). Totally, 95 human thyroid tissues were obtained from fresh frozen surgically resected thyroid tissues (≥15 mm). In order to reduce contamination, all resected tissues were snap frozen in liquid nitrogen and independently analyzed. The demographic information of the patients and adenoma characteristics analyzed in this study are presented in Table 1. An informed consent was taken from all participants for the tissue collection in compliance with our institutional guidelines.

**DNA extraction**

Fresh frozen tissue specimens were cut on dry ice from fresh frozen surgical material stored at −180°C by using a scalpel. Then, DNA was extracted using the DNeasy Blood & Tissue Kit (Qiagen, Cat No:69504) according to the manufacturer’s protocol. DNA purity and quantity was determined using a Thermo Scientific™ NanoDrop™ spectrophotometers 2000c spectrophotometer (Thermo Fisher Scientific Inc). All the extracted thyroid tissue DNAs were stored at - 80^°^C. 

**Table 1:** Demographics of PTC patients (cases) and goiter patients (control)

Variables	Case (n= 55)	Controls (n=40)	P-value
Age (years)	42.28 (±14.32)	43.16 (±9..31)	0.624
Gender			
Female	38 (70.90%)	31 (77.50%)	
Male	17 (30.90%)	9 (22.5%)	0.306
Weight	70.51 (±12.19)	73.60 (±10.91)	0.204
Height	164.96 (±7.96)	167.32 (±9.55)	0.370
BMI	28.85 (±4.09)	31.26 (±0.79)	0.093
SBP	121.91 (±16.01)	118.77 (±11.10)	0.963
DBP	76.58 (±11.79)	70.05 (±9.79)	0.491

**BMI**: Body Mass Index, **SBP**: Systolic Blood Pressure**, DBP**: Diastolic Blood Pressure.

There was no statistically significant difference between the two groups of PTC patients (cases) and goiter patients (control) in basic characteristics.

**Bisulfite Modification** **and Quantitative Methylation Detection**

Bisulfite conversion altered the DNA sequence depending on the methylation status of individual unmethylated Cytosines (C) conversion to uracil in genomic DNA; these changes can be detected via HRM analysis (54). Then, 150-200 ng of DNA from each sample was treated with sodium bisulfite conversion kit by the “EpiTect® Bisulfite Kit” (Qiagen, Cat No:  59104) according to the manufacturer's protocol. The melting profiles of bisulfite-modified PCR products can be used to indicate methylation status, when samples are unknown. These processes were run by bisulfiting pre-treatment and unbiased PCR amplification of both methylated and unmethylated templates of the targeted region. Therefore, for the MS-HRM of *SLC5A8, RASSF1, MGMT *and *DNMT1* genes promoter region amplification we used gene specific primers. The MS-HRM analyses were run based on three main stages: holding stage, cycling stage, and melt curve stage.

**Statistical Analysis**

Samples were considered as hyper-methylated and hypo-methylated when the measured methylation point was more than the 12% mean methylation level and less than the 12% mean methylation level, respectively. Correlations between the methylation levels and demographic and histopathological characteristics in the two groups were analyzed using DNA methylation. All analyses were done by SPSS^®^, version 16.0, license (SPSS Inc., Chicago, IL. USA), and P-value<0.05 was considered as statistically significant.

## Results

**Fig. 1:**
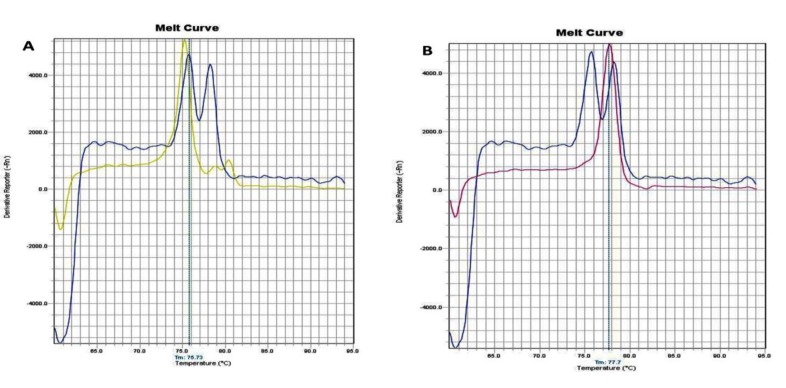
The graph of targeted promoter region of RASSF1 in comparison with 50% controls. Part A indicates hypo-methylation (yellow line) and Part B indicates hyper-methylation (red line)

We examined the 12-loci promoter methylation of fresh frozen tissue (55 PTC cases vs. 40 goiter controls) for four tumor suppressor genes. The age of all samples ranged from 18 to 86 years. For each run of MS-HRM, five wells were allocated to control samples (0%, 50%, and 100%). Numerous replicates of the diluted samples were amplified by PCR. Then melting profile of each reaction was used to define its methylation status. The hyper/hypo-methylations in each sample could be freely estimated by comparing them to the control peaks (Figure 1).

Quantification of each locus of the four targeted genes is presented in Table 3. In addition, each locus methylation quantification and the overall methylation status of each gene was earned through two regions of *RASSF1*, three regions of *SLC5A8*, three regions of *DNMT1,* and four regions of *MGMT* (Table 2).

The cut-off value of methylation was defined according to several references (U if it was ≤12% and M if it was ≥12%) (55-57).

All candidate tumor suppressor gene promoter hyper-methylation was significantly different in two groups of PTC cases and controls except *DNMT1*. Thus, for sensitivity analysis adjusting for demographic variables and risk factors we have done several logistic regression models for potentially confounding variables Table 3). Two DNA methyltransferases of *MGMT* and *DNMT1* also adjusted in model III and IV in order to check their impact on the methylation pattern.

In model III (*MGMT* adjusted model) the odds ratio of *RASSF1* and *SLC5A8 *promoter hyper-methylation and risk of PTC increased in comparison with model I (crude model). However, in model IV (*DNMT1* adjusted model), the *RASSF1* and *SLC5A8* promoter hyper-methylation and risk of PTC decreased a little in comparison with model I (crude model).

**Table 2:** The difference of methylation quantification of 12-promoter loci of four targeted tumor suppressor genes

Promoter Region	Methylation*	PTC Cases Number (percent)	Goiter Cases Number (percent)	P-value
*SLC5A8*	U	16(29.09%)	34(85.0%)	0.002
	M	39 (70.9%)	8 (20.0%)	
*RASSF1*	U	11(20.8%)	38 (95.0%)	<0.001**
	M	44(80.0%)	2 (5.0%)	
*MGMT*	U	7 (14.0%)	29 (72.5%)	0.001
	M	49 (12.72%)	11 (27.5%)	
*DNMT1*	U	25 (45.45%)	30 (75%)	0.018
	M	30 (54.54%)	10 (25%)	

*Methylation is categorized from 1 to 5 according to methylation quantification results.

1=0 percent methylated; 2=25 percent methylated; 3=50 percent methylated; 4=75 percent methylated; and 5=100 percent methylated.

The mean methylation of several loci of one gene was calculated and categorized as U if it was ≤12% and M if it was ≥12%** The most significant P-value was reported for *RASSF1* gene.

**Table 3:** The association of methylation in four tumor suppressor genes *SLC5A8*, *RASSF1*, *MGMT,* and *DNMT1*

Model	*SLC5A8*	*RASSF1*
Model I	8.24 (3.34 - 20.32)	18.37 (6.65 - 50.76)
Model II	8.72 (3.36 - 22.67)	16.29 (5.72 - 46.34)
Model III	9.94 (2.87 - 34.45)	19.02 (4.97 - 75.52)
Model IV	8.85 (3.23 - 24.24)	16.52 (5.62 - 48.6)

Model I is crude model, Model II is age and sex adjusted model, Model III is age, sex, and *MGMT* methylation status adjusted, and Model IV is age, sex, *DNMT1* methylation status adjusted.

**Fig. 2:**
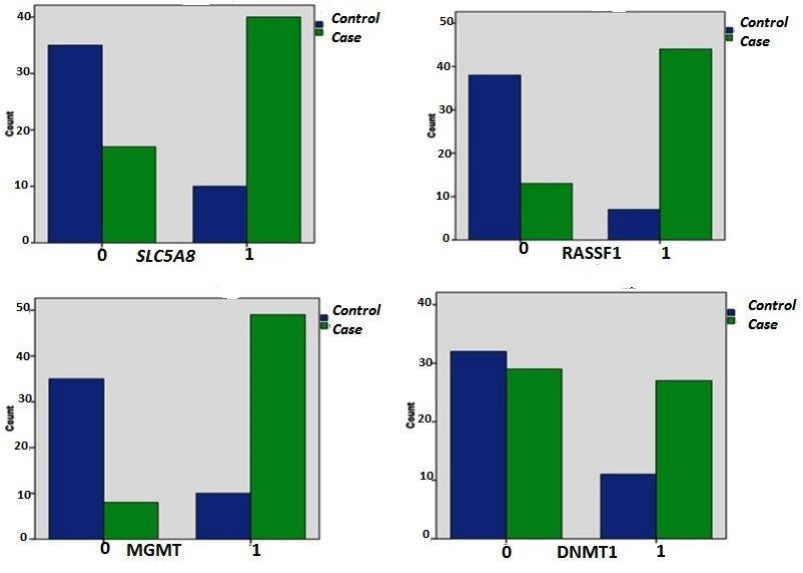
Promoter quantification differences of *SLC5A8, RASSF1, MGMT* and *DNMT1 *methylation in PTC cases and controls

## Discussion

The role of aberrant methylation of tumor suppressor genes more than functionally chief regulatory genes is a frequent event in certain human tumors and developmental abnormalities (58, 59). In eukaryotic cells there are special DNA methyltransferase enzymes which put or remove the methyl group on/off the cytosine base of CpG islands and regulate the conformation change between methylated and unmethylated forms (60, 61). *DNMT1* is the most abundant DNA methyltransferase in mammalian cells and extra active on hemi-methylated DNA as compared with unmethylated substrate in vitro, but it is still more active at de novo methylation than other *DNMTs* (62-64). Several studies have shown that changing the DNA methylation patterns in the regulatory promoter regions of *DNMT1* play an important role in the development of genetic disorders (65-68). *DNMT1* promoter methylation was present in primary and recurrent gliomas (69). The *DNMT1* expression can be regulated through its promoter methylation patterns in the core promoter region of *DNMT1* in several human neoplastic tissues (70, 71). The different methylation status of *DNMT1* (b) in our study can be attributed to this region as the core promoter region that can have a role in PTC. It can also be supported by the idea that epigenetic regulation of the methylation status of *DNMT* genes can regulate epigenetic profile of extra embryonic tissue in humans (72). It has been shown that some medication targeting *DNMT1* can inhibit migration and invasion of thyroid cancer cells through down-regulating *DNMT1* (73). In contrast, some results indicated that *DNMT1* was neither overexpressed in PTC nor correlated with tumor stage and capsular/vascular or lymphatic invasion (74). Yi Cai *et al.* (2017) pointed out to the critical threshold levels of *DNMT1* as an important factor of DNA methylation maintenance across the genome in human cancer cells (75). However, our results indicated that *DNMT1* methylation in PTC patients, in comparison with goiter patients (controls), was less than *SLC5A8*, *RASSF1*, and *MGMT*. 

O^6^-methylguanine DNA methyltransferase is a protein in humans encoded by the *MGMT *gene, and it is a maintenance methyltransferases that is crucial for genome stability (76, 77). In a colorectal cancer study it was suggested that *MGMT* expression reduced after hyper-methylation of the *MGMT* promoter region (78). *MGMT *is suppressed epigenetically and in different ways such as promoter region hyper-methylation and over-expression of a number of microRNAs (79-84). Inactivation of the *MGMT* through promoter hyper-methylation is a common event in primary human malignancies (71, 85). Esteller* et al.* described a straight line of *MGMT* aberrant methylation and *k-Ras* and *p53* genes mutation in colorectal cancer (85, 86). The promoter methylation of two candidate regions of *MGMT *were associated with PTC. In a *MGMT* adjusted model the odds ratio of *RASSF1* and *SLC5A8* promoter hyper-methylation and risk of PTC increased in comparison with model I (crude model). Supporting information is reported by Herfarth *et al.* that linked the specific CpG methylation pattern of the *MGMT* promoter region with decrease of *MGMT* expression in primary colorectal cancers (87). Moreover *MGMT* methylation is reported in a group of discriminating methylation markers that differentiate thyroid cancer from benign nodules (88). Moreover, *MGMT* methylation was reported in *MLH1* and *MGMT* expression and their consequence in genomic instability in patients with thyroid carcinoma (89).

*SLC5A8* can predominantly be found in the small intestine, colon, thyroid gland, kidney, and salivary glands and to a lesser extent in the retina and brain (90-93). It was shown that *SLC5A8* expression, as a sodium/iodide symporter (NIS) member, decreased in several malignancies, including thyroid cancers and its methylation is shown as the discriminative marker between malignant and benign thyroid tumors (88, 94). We recently conducted a meta-analysis the results of which indicated that* SLC5A8* was the most significant methylated gene in thyroid cancers (95). CpG island methylation of tumor-related promoters including *RASSF1, MGMT, *and *SLC5A8* occurs preferentially in undifferentiated thyroid carcinoma (96). In contrary, the overexpression of *SLC5A8* together with *IRX1* and *EBF3* may be involved in the transforming growth factor beta signaling pathway, which is often disrupted in head and neck squamous cell carcinoma. Silencing of the *SLC5A8* through its promoter methylation was associated with BRAF mutations in classical PTC (97). Hyper-methylation of *SLC5A8* promoters reported preferentially in undifferentiated carcinoma (98). 

The *Ras association domain-containing protein 1 (RASSF1), *which is encoded by *RAS *gene altered expression, is associated with the pathogenesis of a variety of cancers (99-103). The most frequent molecular mechanism for *RASSF1* suppression in different malignancies is the ishypermethylation of its CpG-island promoter region (104-108). Frequent epigenetic silencing of the *RASSF1A* in thyroid carcinoma has been highlithed (109) alone or with *NORE1A* methylation and *BRAF*^V600E^ mutations (110). *RASSF1 *methylation can be used as the therapeutic determinant in thyroid malignancies (111). In a meta-analysis it was reported as the most significant hyper-methylated region within thyroid carcinomas (112). The survival rate and prognosis in head and neck squamous cell carcinoma (HNSCC) patients was dependent on 11 tumor-related genes, including *RASSF1* and *MGMT*. The adjusted model of age, sex, and *DNMT1* methylation status did not change the link of *RASSF1* methylation and PTC cancer risk. Meanwhile, Bai *et al*. reported that *DNMT1* inhibits proliferation, metastasis, and invasion in esophageal squamous cell carcinoma by suppressing methylation of *RASSF1* and *DAPK* (30, 113). Methylation of *RASSF1* gene promoter can be regulated by *p53* and *DAXX* (114, 115).

According to the results, *RASSF1 and SLC5A8* promoter methylations can be a PTC diagnostic biomarker which are completely dependent on *DNMT1* or *MGMT* promoter methylation status.
